# Reduction in Toxicity of Nano-Ag-Polyvinyl-pyrrolidone Using *Hydra* Proteins and Peptides during Zebrafish Embryogenesis

**DOI:** 10.3390/nano9091210

**Published:** 2019-08-27

**Authors:** Soon Seok Kim, Jin Ah Lee, Min-Kyeong Yeo

**Affiliations:** Department of Environmental Science and Engineering, College of Engineering, Kyung Hee University, 1732 Deogyeong-daero, Giheung-gu, Yongin-si, Gyeonggi-do, Seoul 17104, Korea

**Keywords:** nanotoxicity, nano-Ag-polyvinylpyrrolidone, *Hydra* protein, *Hydra* peptide, reduced toxicity, microarray

## Abstract

*Hydra magnipapillata* cells reduce the toxicity of silver nanomaterials to zebrafish (*Danio rerio*) embryos. In this study, we investigated whether *Hydra* protein (HP) and *Hydra* basal disc peptide (Hym176) materials reduce nano-Ag-polyvinylpyrrolidone (N-Ag-PVP) toxicity during embryogenesis of the nanosensitive organism zebrafish. Protein (HP) was extracted from *Hydra*, and peptide (Hym176) was extracted from the hydra basal disc, which is attractive to nanomaterials and related to the immune system. The experimental conditions were exposure to N-Ag-PVP, HP, N-Ag-PVP+HP, Hym176, or N-Ag-PVP+Hym176 during embryo development. N-Ag-PVP+HP group showed lower toxicity than N-Ag-PVP group. In addition, in the N-Ag-PVP+HP group formed aggregated nanomaterials (≥200 nm size) through electrostatic bonding. In the gene expression profile, HP group differed in gene expression profile compared the other experimental groups and it was no genetic toxicity. HP showed a tendency to reduce side effects and abnormal gene expression produced by N-Ag-PVP with no evidence of inherent toxicity. Considering the potential nanotoxicity effects of released nanomaterials on the ecosystem, the reduction of nanotoxicity observed with HP natural materials should be regarded with great interest in terms of the overall health of the ecosystem.

## 1. Introduction

The chemical composition, morphology, density, and physicochemical properties of nanomaterials differ from those of conventional bulk materials [1]. Among the commercially available nanomaterials, silver nanomaterials exhibit high antimicrobial activity [2], are incorporated into antimicrobial plastics, fabrics [3], fibers [4], food supplements, and packing [5], and are used as water disinfectants and to improve wound healing [6]. However, many studies have also reported toxicity caused by silver nanomaterials, including cellular DNA damage, protein oxidation, production of reactive oxygen species (ROS), and induction of cell damage and death [2]. The toxicity of silver nanomaterials was previously reported in a study in which zebrafish embryos were exposed to treated water [7,8].

Owing to their widespread use, silver nanomaterials are now dispersed throughout the environment, and both humans and ecological organisms are widely exposed to them [9,10]. Therefore, there is an increasing need to investigate the ecotoxicity of silver nanomaterials and manage those risks within a regulatory framework for use of silver nanomaterials [11]. Nanomaterials directly introduced into rivers can endanger the ecosystem and human health [12], nanomaterials that have not been removed during wastewater treatment may enter and affect river ecosystems [13,14]. Degradation of nanomaterials after use is necessary to prevent their release into ecosystems. However, chemical-based destruction can cause the release of secondary pollutants such as trihalomethanes (THM) [15]. Therefore, there is a need for treatment methods that use bio-based natural substances.

In our previous study, exposure to 100 mg/L of silver nanocolloids produced limited toxicity in *Hydra magnipapillata*, which showed 100% regeneration after 7 days [8]. When zebrafish were exposed to *Hydra* cells mixed with silver nanocolloids and nanotubes, the hatching percentage increased, the abnormality percentage decreased, and the expression of apoptosis-related genes was reduced compared to embryos exposed only to the silver nanocolloids and nanotubes [16]. The silver concentrations used in the *Hydra* cell co-exposure study were 50,000–100,000-fold greater than the concentrations that decreased the hatching percentage and induced abnormalities in zebrafish exposed to nanosilver alone (nanosilver 20 ng/L and silver ions 10 ng/L) [17]. However, the survival and regeneration percentages of the exposed *Hydra* and the shape and size of the agglomerated nanomaterials in the basal disk of *Hydra* were changed after exposure to nanosilver. Unlike vertebrates, *Hydra*, a Cnidarian, has only an innate immune system. The *Hydra* basal disc is associated with defense and immune functions and contains the bulk of the antimicrobial peptides (AMP) the organism uses to defend against external bacterial attacks [18]. The role of the basal disc in defense against nanomaterial exposure therefore needs to be investigated [19,20,21]. Hym176 is one of neuropeptides on epithelial cells and neuropeptides of *Hydra* were found to have indeed antimicrobial activity [21]. Silver nanomaterials were aggregated at the *Hydra* basal disc [8] that consists of Hym176 neuropeptides.

In this study, we based on the results of a previous study in which *Hydra* were not affected by the toxicity of nanomaterials [8], and we used *Hydra* protein (HP) and *Hydra* basal disc peptide (Hym176) preparations as biobased substances to reduce the toxicity of N-Ag-PVP. The physicochemical properties of nano-Ag-polyvinylpyrrolidone (N-Ag-PVP) were analyzed in two types of biomaterial (HP, Hym176)-containing environments.

Zebrafish embryos were then co-exposed to the nanosilver and/or *Hydra* preparations to test their cytotoxicity. The percentages of abnormal hatched larvae and larval morphology were assessed 72 h post-fertilization (hpf). Gene expression levels in the hatched larvae were confirmed by mRNA-seq analysis and quantified using quantitative real-time polymerase chain reaction (qRT-PCR).

## 2. Materials and Methods

### 2.1. Preparation of N-Ag-PVP, HP, and Hym176

N-Ag-PVP was purchased from Avention Co. (Seoul, Korea). The N-Ag-PVP powder was mixed with distilled water and dispersed using an ultrasonic generator (Power Sonic 405, Hwashin Tech, Seoul, Korea) for 40 min at 19 ± 1 °C to prepare a stock solution, which was then diluted with dechlorinated tap water to yield a final concentration of 1 mg/L. Immediately before exposure, the solution was dispersed again for 40 min using an ultrasonic generator.

Hydra protein was extracted using TRIzol™ (Invitrogen Co., Wilmington, DE, USA) with some modifications. Using 100 mg of *Hydra* which had been pulverized, then 200 μL of Red Orange was fractionated. Final step, the HP stored in distilled water at 4 °C, and used for experiments within 24 h. Distilled water was removed, the HP was crushed with a homogenizer. Dechlorinated tap water was added to the crushed HP to yield a final concentration of 4 mg/L, which was then dispersed using an ultrasonic generator at 19 ± 1 °C for 80 min.

The *Hydra* peptide Hym176 (H-Ala-Pro-Phe-Ile-Phe-Pro-Gly-Pro-Lys-Val-NH_2_) was synthesized using the method proposed by Fujisawa [22] with some modifications. First, 0.637 g of rink amide AM resin was dissolved in dichloromethane (DCM) and allowed to swell for 30 min. The resin was then mixed with deprotected fluorenylmethyloxycarbonyl (Fmoc) for 10 min and washed with 20% piperidine/dimethylformamide (DMF). Resin coupling was carried out at 22 ± 1 °C for 40 min, and then 0.2 mmoL Fmoc-Val-OH, 0.2 mmoL 2-(1*H*-benzotriazole-1-yl)-1,1,3,3-tetramethylammonium tetra-fluoroborate (TBTU), and 0.2 mmoL *N*,*N*-diisopropylethylamine (DIEA) were added to the resin. After coupling, the resin was washed one or two times with DMF. This process was repeated until all the amino acids were continuously coupled to the chain, extending the length of the peptide. When the final amino acid was coupled to the chain, the de-protected Fmoc-treated peptide was washed three times with methanol and then dried. The dried resin was added, and an appropriate amount of cleavage solution was added before incubation (40 °C, 3.5 h). The solution was filtered, ether was added to the precipitate, and centrifugation (1006.2 *g*, 2 min) was performed twice. The synthesized Hym176 was dried for several minutes, lyophilized, and then stored at −20 °C. The lyophilized Hym176 was dissolved completely in 1 mL of distilled water, diluted with 4 mg/L of dechlorinated tap water, and then dispersed using an ultrasonic generator at 19 ± 1 °C for 80 min.

### 2.2. Physicochemical Properties of N-Ag-PVP and N-Ag-PVP+HP

Field emission scanning electron microscopy (FE-SEM, LEO SUPRA 55, Carl Zeiss, Oberkochen, Germany; 10 kV) was used to assess the surface morphology of N-Ag-PVP and N-Ag-PVP+HP (Figure S1A). The crystallinity of N-Ag-PVP and N-Ag-PVP+HP was analyzed using an X-ray diffractometer (XRD, model PW 1830, Philips, Amsterdam, The Netherlands; 40 mA, 40 kV, 3–90, 6 deg/min). A nickel filter and CuK radiation (30 kV, 30 mA) were used, and the analyses were performed within a 2θ range of 5–80° at a scan rate of 10°/min (Figure S1B). The material distribution characteristics and zeta-potential were measured using a particle size analyzer (ELS-Z2, Tokyo, Japan). Analytical samples were diluted with distilled water. The physical conditions were as follows: water temperature 25 °C, refractive index 1.3328, and viscosity 0.8878 centipoise (cP).

### 2.3. Experimental Animals

The zebrafish (*Danio rerio*, wild type) used in this study were grown in our laboratory to 7–8 months of age. The zebrafish were reared according to the method proposed by Westerfield [23], using the following conditions: water temperature 28 ± 1 °C; light/dark photoperiod 14/10 h; pH 7.1 ± 0.5; chlorine concentration 0.8 mg/L; ionic strength 2.2 × 10^5^; and total organic carbon (TOC) concentration 0.9 mg/L. The fish were fed brine shrimp (*Artemia* sp.), bloodworms, and dry flake feed (TetraMin, Melle, Germany) three times a day.

The *Hydra* were obtained from the Korea Ocean Research and Development Institute (KORDI, Geoje, Korea). The *Hydra* were incubated in 0.5 L of *Hydra* culture medium at a water temperature of 20 ± 1 °C with a photoperiod of light/dark 16/8 h [24,25]. All animal experiments were approved by the Institutional Animal Care and Use Committee (IACUC) of Kyung Hee University and were conducted according to the provisions of the IACUC.

### 2.4. Exposure Conditions

The N-Ag-PVP, HP, and Hym176 preparations were dispersed immediately before exposure. The N-Ag-PVP+HP and N-Ag-PVP+Hym176 were dispersed independently using an ultrasonic generator for 40 min, mixed, and dispersed for an additional 40 min.

The collected embryos were washed with dechlorinated tap water at 20.5 °C, after which 20 embryos were allocated to each well of 6-well plates. In the experimental groups, 60 embryos were exposed to N-Ag-PVP (1 mg/L), HP (4 mg/L), N-Ag-PVP+HP (1 or 4 mg/L), Hym176 (4 mg/L), or N-Ag-PVP+Hym176 (1 or 4 mg/L). The 4 mg/L HP concentration was chosen based on the results of prior range-finding experiments. All experimental groups were exposed simultaneously for 1 hpf to ensure the same embryonic stage across treatments. Subsequently, the embryos were observed using a stereoscopic microscope (SZ61, Olympus, Tokyo, Japan) at 4, 12, 24, 32, and 48 h after exposure [16,17]. Embryonic staging was carried out according to the standardized staging series established by Kimmel et al. [26]. The exposed embryos were maintained at 28 ± 1 °C in an incubator. Dead embryos were removed to avoid contamination of the test solution. After 72 h of exposure, embryos that did not hatch were classified as dead, and the hatching percentage, abnormality percentage, and morphological abnormality percentage of viable larvae were assessed. The experiment was repeated three times, and the values are expressed as mean ± standard deviation (SD).

### 2.5. Microarray Analysis

Total RNA was isolated from the hatched zebrafish larvae (72 hpf, whole body exposure) using TRIzol™ (Thermo Fisher Scientific, Wilmington, DE, USA ). The quality of the RNA was assessed with an Agilent 2100 bioanalyzer (Agilent Tech., Waldbromm, Germany) using an RNA 6000 Nano Chip (Agilent Technologies, Amstelveen, The Netherlands). The RNA was quantified using an ND-2000 spectrophotometer (Thermo Fisher Scientific, Wilmington, DE, USA ) and confirmed to have an absorbance ratio >1.8 and integrity >7.0.

The SENSE mRNA-Seq library preparation kit (Lexogen, Inc., Vienna, Austria) was used for mRNA sequencing and library construction. High-throughput sequencing was performed using a HiSeq 2000 (Illumina, Inc., San Diego, CA, USA). The library was prepared from a 2 μg sample of total RNA using the SMARTer Stranded RNA-Seq Kit (Takara Bio, CA, USA). Separation of mRNA was performed using a Poly (A) RNA selection kit (LEXOGEN, Inc., Vienna, Austria). Isolated mRNA was synthesized into cDNA and sheared according to the manufacturer’s instructions. Indexing was performed using Illumina index 1–12. Polymerase chain reaction was used for amplification. The library was identified using the Agilent 2100 bioanalyzer (DNA High Sensitivity Kit), and the average fragment size was assessed. Quantification was performed using (Thermo Fisher Scientific, Wilmington, DE, USA) and a library quantification kit. High-throughput sequencing was performed with 100-bp paired-end sequencing using a HiSeq 2500 (Illumina, Inc.).

To read the mRNA-seq, mapping was performed using TopHat to obtain sorted files. Differentially expressed genes (DEG) were determined based on the coefficients of inherent and multiple alignments using *bedtooLs*. The read count data were processed based on the quantile normalization method, using EdgeR in R (R development Core Team, 2016) and Bioconductor. The sorted files were also used to combine copies, estimate abundances, and detect DEG or isoforms using Cufflinks. Fragments per kilobase of exon per million fragments (FPKM) were used to determine the expression levels of the genes.

Up- or down-regulated genes were identified using ExDEGA v1.2.1.0 (EBIOGEN, Inc., Seoul, Korea). Categorization of the genes was based on a search performed using DAVID (http://david.abcc.ncifcrf.gov). In each experimental group, FPKM gene expression level was converted to a log2 value, and the relative level with respect to the control group is presented. Approximately 3000 genes selected from the DEG analysis were analyzed by DAVID. The Gene Ontology (GO) from the DAVID analysis was used to show the correlation in Quick GO (https://www.ebi.ac.uk/QuickGO). The genes involved in tight junctions, forkhead box O (FOXO) and mitogen-activated protein kinase (MAPK) signaling pathways, and G protein-coupled receptors (GPCRs), which affect the embryonic development stage in the KEGG pathway (https://www.genome.jp/kegg/pathway.html), were identified. The clustering heatmap profiles of DEGs were compared across the experimental groups using the Multiple Experiment Viewer software program v4.9 (MeV). The average fold-change (FC) for each gene was expressed as a standardized z-score.

### 2.6. qRT-PCR Analysis

Table 1 summarizes the primer information for the seven major genes (*cldna*, *cdc42l*, *igf1*, *zgc:55558*, *mapk10*, *mapk11*, and *tp53*) in the signaling pathway of the cells used for qRT-PCR and that of the reference gene (*actb2*). Claudin is a key component of tight junctions and maintains intercellular junctions, while some claudin gene defects have been reported to cause hearing loss [27]. Cdc42l is a Ras-related GTP-binding protein that is required for angiogenesis by regulating the filament-actin (F-actin) of filopodia [28]. In the MAPK signaling pathway, Igf1 plays an important role in embryonic growth and development [29]. zgc:55558 (Ras), mapk10 (JNK), mapk11 (ERK), and tp53 (p53) are located in the main pathway that receives extracellular signals from growth factors and integrates signals, thus promoting cell growth and proliferation [16].

Reverse transcription was performed using 3 μg of total RNA and SuperScript II RTase (Invitrogen, Carlsbad, USA). qRT-PCR was performed in a SuperScriptTM real-time PCR system (Applied Biosystems, Waltham, USA) using a SYBR green PCR kit (Applied Biosystems). The qRT-PCR cycle conditions were as follows: pre-denaturation at 95 °C for 10 min, followed by 40 cycles of 95 °C for 15 s and 59 °C for 30 s. Data were analyzed using StepOne™ software v2.2.2 (Applied Biosystems). The mRNA expression levels for the seven genes were normalized to that of the reference gene and quantified using the 2^−ΔΔCt^ method. Amplification plots and melting curves were obtained for the amplified products.

### 2.7. Statistical Analysis

Statistical analyses were performed using IBM’s Statistical Package for the Social Sciences (SPSS) v23.0 (IBM, Armonk, New York, USA). Analysis of variance (ANOVA) was performed to compare and evaluate the experimental groups, followed by Tukey’s post-hoc test for multiple comparisons. Values were considered statistically significant at *p*-values <0.05 and <0.01.

## 3. Results and Discussion

### 3.1. Properties of N-Ag-PVP and HP

Low organic matter is favorable to NP aggregation in water [30]; thus, all AgNPs particle sizes at 1 h exposures for analysis in distilled water were increased (Table 2). The particle size of N-Ag-PVP (30 nm to 285.50 nm) at 1h was increased by 9 fold more than those on original particle (Figure S1A). In addition, the changes of N-Ag-PVP+HP particle distributions were twice (534.4 nm) than N-Ag-PVP (285.50 nm) because HP seemed to increase aggregation. The nanomaterials were found to have 22.84 (N-Ag-PVP) and 5.54 (N-Ag-PVP+HP) mv of negative charge on the surface. (Table 2). The nanomaterials could have a high affinity for the positively charged molecules in an organism [30], but *Hydra* protein could cause low affinity because it changes the surface charge.

The XRD patterns of N-Ag-PVP and N-Ag-PVP+HP were not different, but the sensitivity with HP was lower. HP did not affect the crystallographic planes of Ag-PVP (Figure S1B). N-Ag-PVP showed peaks at 38°, 44.18°, 64.39°, and 77.32°, while N-Ag-PVP+HP showed peaks at 38.16°, 44.34°, 64.48°, and 77.44°, the characteristic peaks of Ag-PVP [31].

HR-Raman analysis showed different positions of major bands for N-Ag-PVP and HP (Figure S1C). The major band positions in HP were 563, 785, 955, and 1089 cm^−1^. For both the N-Ag-PVP and N-Ag-PVP+HP aggregates, the 236 cm^−1^ and 222 cm^−1^ bands appeared to be due to the Ag-O bonds [32]. Additionally, the C=O and CH_2_ bonds of PVP indicated interaction between PVP and Ag NPs. Analysis of the N-Ag-PVP sample indicated that the 1601 cm^−1^ and 2933 cm^−1^ bands corresponded with C=O and CH_2_ bonds, respectively, while the C=O and CH_2_ bonds corresponded with the 1605 cm^−1^ and 2918 cm^−1^ bands, respectively, for the N-Ag-PVP+HP sample [33].

PVP-coated silver nanoparticles can become stably dispersed in fresh water and may remain dispersed over a wide range of pH values [34]. However, part of the dispersant in the environmental medium can be detached and flocculate or precipitate as a result of displacement by other molecules such as inorganic ions [35,36].

The toxicity of silver nanomaterials is known to be associated with release of silver ions dispersants [37]. Silver ions (Ag^+^) are toxic due to their strong affinity for thiol and disulfide groups [9]. Of note, PVP-coated silver nanomaterials have been reported to release 48% of their silver ions after 120 h in fresh water [38].

### 3.2. Effects of HP and Hym176 on N-Ag-PVP-Exposed Zebrafish Embryos

Approximately 10 min after HP- and N-Ag-PVP+HP-exposure on embryo (1.5 hpf), HP was adsorbed onto the chorion surrounding the embryo (Figure S2c,d). Aggregates, which were not observed in the N-Ag-PVP- and Hym176-exposed groups (Figure S2e), were formed and adsorbed onto the chorion in the N-Ag-PVP+Hym176-exposed group (Figure S2f).

**Figure 1:**
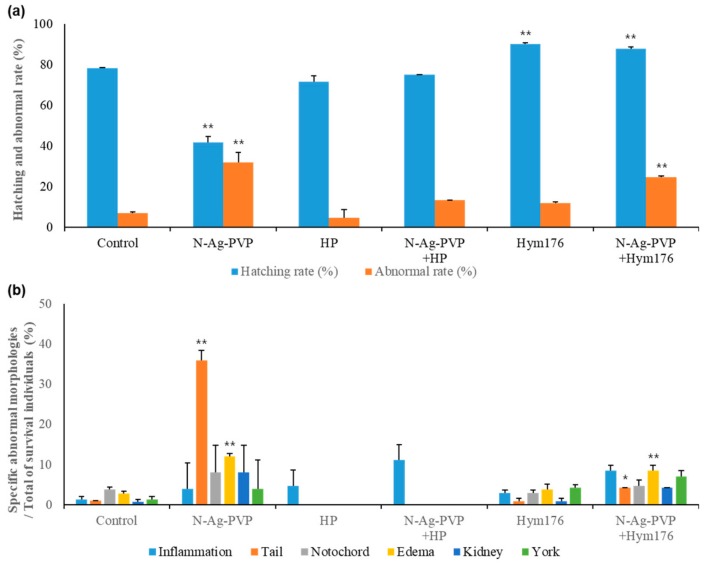
The effects of N-Ag-PVP, HP, N-Ag-PVP+HP, Hym176, and N-Ag-PVP+Hym176 on the hatching and abnormal rate (**a**) and rates of specific abnormal morphologies among surviving embryos (**b**). All measurements are reported as the group mean of three replicates and the standard deviation (n = 60). * comparison of experimental groups to a homogeneous subset of post-hoc test results (*p* < 0.05), ** comparison of experimental groups to a homogeneous subset of post-hoc test results (*p* < 0.01).

The results of the zebrafish embryo exposures are presented as hatching percentages, abnormality percentages, and morphological abnormality percentages. Significance was assessed using a homogeneous subset of post-hoc test results. The hatching percentage of the N-Ag-PVP-exposed group was 41.7 ± 2.9%, which was significantly lower than that of the control group (78.3 ± 0.4%; *p* < 0.01; Figure 1a). The hatching percentages of the HP- and N-Ag-PVP-HP-exposed groups were similar to that of the control group. On the other hand, the abnormality percentages of the N-Ag-PVP- and N-Ag-PVP+Hym176-exposed groups significantly increased to 4.0- and 3.53-fold greater that of the control group, respectively (*p* < 0.01). In N-Ag-PVP-exposed embryos, tail morphogenesis was abnormal in 36% of the hatched larvae (*p* < 0.01; Figure 1b). Overall, the N-Ag-PVP- and N-Ag-PVP+Hym176-exposed groups had abnormal tails and edema.

Complex abnormalities were observed in larvae exposed to N-Ag-PVP. Edema of the heart and yolk sac, curved notochord loss, inflammation of the tail and fins, and abnormal kidney morphology were observed (Figure S3b). Fin inflammation was observed in the HP- and N-Ag-PVP+HP-exposed groups (Figure S3c,d). In the Hym176-exposed group, complex abnormalities (inflammation, edema of the head, heart and yolk sac, abnormal kidney morphology and bleeding, and curved notochord) were identified (Figure S3e). Lastly, edema and a cracked yolk sac surface, inflammation, kidney morphological deformities, and a curved notochord were evident in the N-Ag-PVP+Hym176-exposed group (Figure S3f). Overall, the Hym176- and N-Ag-PVP+Hym176-exposed groups showed abnormalities such as inflammation of the embryo, curved notochord, and edema in major organs, similar to the N-Ag-PVP-exposed group.

The toxicity of silver nanomaterials in the embryo is likely related to their adsorption onto the cell membrane [38]. The aggregates of N-Ag-PVP and HP were greater than 200 nm in size and could not easily pass through the chorion pore canals (CPCs), thereby reducing the effects of N-Ag-PVP exposure on zebrafish embryos [39]. When present within the chorion (thickness = 3.5 μm, CPC diameter = 0.5–0.7 μm, and pore spacing = 1.5–2.5 μm) and around the embryo, nanoparticles ≤ 100 nm in size may penetrate the CPCs in a concentration-dependent manner, increasing internal exposure and consequent toxicity to the embryo [40]. As the aggregates formed between N-Ag-PVP and Hym176 (30 base pair (bp), 11–12 nm) were relatively small compared with the HP aggregates, they are likely to penetrate directly through CPCs. The absorption of nanomaterials by endocytosis is more a function of particle size than of chemical properties, with a 100 nm upper limit for effective absorption through the cell membrane. Silver nanomaterials may be adsorbed onto the cell membrane, subsequently reaching the nucleus by endocytosis [41].

We postulated that HP and Hym176 may bind to and consequently reduce the toxicity of silver nanomaterials dispersed in water. In our pre-experiments, we found that the toxicity of N-Ag-PVP (1 mg/L) was reduced by co-incubation with HP (2, 4, or 6 mg/L) in a concentration-dependent manner. This result is similar to previous results indicating that the Ag ion of Ag NP is bound by natural organic matter, such as humic acid, in natural waters [42]. In many commonly used test organisms, exposure to nanomaterials produces adverse effects on the immune system, such as apoptosis, but *Hydra* appear to be less sensitive to immune toxicity [8]. We have investigated the possibility of several hydra peptides (Hym323, Hym330, Hym346) that constitute the basal site in consideration of the resistance and regeneration response of Hydra to nanomaterial toxicity [20]. The peptide Hym176 was not difference to the hatching rate of embryos after mixing with N-Ag-PVP but the abnormal rate was increased on N-Ag-PVP+Hym176 exposed group. However, the hatching rates of Hym176 and N-Ag-PVP+Hym176 exposed groups were over 80% (Figure 1). These result based with the Hydra basal disc is associated with defense and immune functions [21] and Hydra basal disc peptide (Hym176) probably defense against nanomaterial exposure. However, there were abnormal phenotypes (edema, tail abnormal) on Hym176 and N-Ag-PVP+Hym176 exposed groups (Figure 1, Figure S2e,f).

There are two possible explanations for the toxic effects of the *Hydra* peptides. The first is the size of Hym176 (30 bp, 11–12 nm), and the second is the effect of the amide functional group at the Hym176 sequence end. It is speculated that aggregates formed between N-Ag-PVP and Hym176 are more likely to penetrate directly through CPCs because they are relatively small compared to aggregated N-Ag-PVP and HP.

However, the peptides consisting only of amino acids did not affect the hatching percentage of the embryos before or after mixing with N-Ag-PVP. The amino group (NH_2_^+^) at the terminal end of Hym176 appeared to be the main binding site for N-Ag-PVP. To reduce the concentration and toxicity of silver nanomaterials in water, it is worth considering the use of organic matter or a substance with a functional group that can induce dislocation.

### 3.3. Microarray and qRT-PCR Analyses

The clustering heatmap profile of DEGs for 10,308 genes in the exposed embryos of each experimental group after mRNA sequence analyses was expressed as a z-score that standardized each average FC value using the MeV program (Figure S4). The mRNA sequence analysis was carried out based on the 14,474 zebrafish (danRer10) genes registered in the University of California Santa Cruz (UCSC) Genome Browser database. The DEG analyses using the ExDEGA program examined a number of genes with FC value ≥2 and ≤0.5 and normalized RC (log2) value ≥10. All treatment groups had more down-regulated than up-regulated genes. Among these comparisons of DEGS, HP vs. control had 2686 up- or down-regulated genes, while N-Ag-PVP+HP vs. HP had the greatest difference in number of differentially expressed genes (2879 genes).

**Figure 2:**
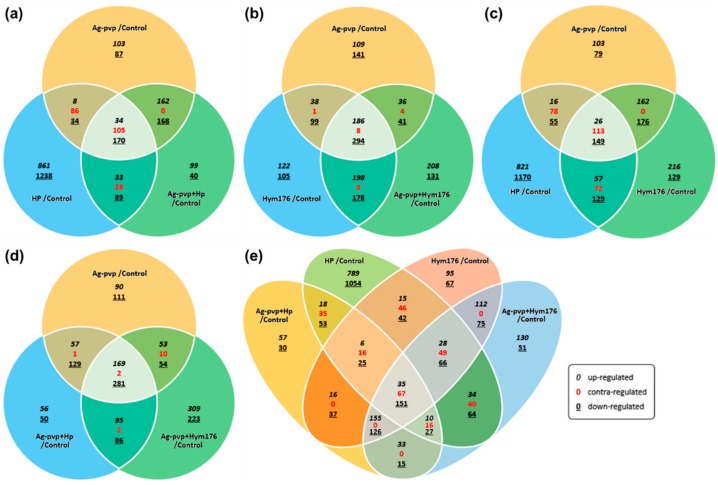
Venn diagrams illustrating the gene expression patterns in each experimental group; contra-regulated means that the effects on gene expression are opposite to each other (Fold change: ≥2, ≤0.5; Normalized RC (log2): ≥10). (**a**)Ag-PVP/control vs HP/control vs Ag-PVP+HP/control, (**b**)Ag-PVP/control vs Hym176/control vs Ag-PVP+Hym176/control, (**c**)Ag-PVP/control vs HP/control vs Hym176/control, (**d**)Ag-PVP/control vs Ag-PVP+HP/control vs Ag-PVP+ Hym176/control, (**e**)Ag-PVP+HP/control vs HP/control vs Hym176/control vs Ag-PVP+ Hym176/control.

The effects of the various treatments on gene expression were determined using Venn diagrams. The HP treatment had an independent effect on 861/1238 genes, the highest among all experimental groups, not while but 33/28/89 (up/contra/down-regulated gene number) genes were affected solely by HP (Figure 2a). In the Hym176-exposed group, the expression of 198/0/178 genes was altered (Figure 2b). Moreover, N-Ag-PVP, HP, or Hym176, when added independently to embryo cultures, affected 26/113/149 genes, respectively (Figure 2c). In the N-Ag-PVP-exposed group, the expression of 169/2/281 genes was altered (Figure 2d). On the other hand, in the four experimental groups, with the exception of N-Ag-PVP, the numbers of genes that showed FC (up/contra/down-regulated gene number) were 35/67/151, which may significantly affect embryogenesis (Figure 2e). However, the presence of 67 contra-regulated genes was likely to have caused errors in the analysis. The contra-regulated gene conflicts with the results of the experimental group but could be an indicator of the tendency for the N-Ag-PVP, HP, and Hym176 exposure groups to effect one other.

Microarray and DEG analyses were performed on the FC data for genes that affect morphogenesis and development of important organs (heart, eyes, nerves, etc.) during early embryonic development (Table 3 and Table 4).

GO analyses revealed that a large number of genes associated with the N-Ag-PVP-exposed group are linked with epistatic interactions during morphogenesis and development. In particular, the GOs associated with morphogenesis via epistatic interactions were animal organ morphogenesis (GO:0009887), anatomical structure morphogenesis (GO:0009653), and developmental processes (GO:0032502). Therefore, gene mutations induced by N-Ag-PVP toxicity may directly cause abnormal appearance of exposed larvae. Moreover, some of the genes involved in morphogenesis and development are involved in abnormalities of important organs and other diseases. In addition to embryonic development, lmo7b, lum, sec23a, fn1b, zeb2b, and fxr1, which were found in various GOs, affect the heart [43], nearsightedness [44], skeletal morphogenesis [45,46], tissue regeneration [47,48] and signal transduction [49,50], thereby inducing weakness, mental retardation [51], and muscular atrophy [52].

Therefore, N-Ag-PVP and Hym176 aggregates, which are small, seemed to influence embryonic organ morphogenesis and development by passing through the embryonic CPCs and cell membrane. It has been reported that N-Ag-PVP, which penetrates the embryos through CPCs, is adsorbed onto the surface of cell membrane proteins, activating them [53]. This interaction may lead to immobilization of cell membrane proteins and up-regulation of GPCR-associated genes [54].

**Figure 3:**
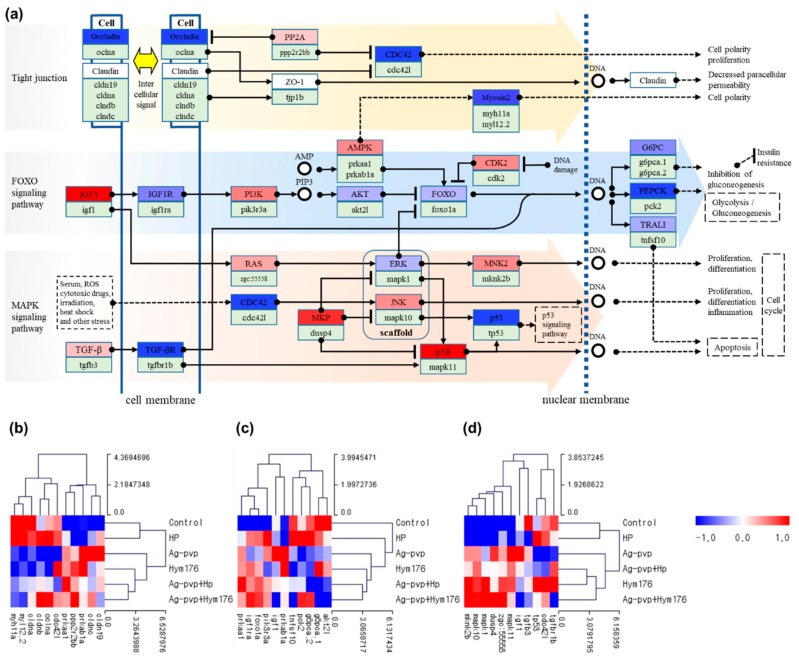
(**a**) Pathway for effects on cell cycle regulation and intercellular signaling in zebrafish exposed to N-Ag-PVP; and heat maps of the (**b**) tight junction, (**c**) fork headbox O (FOXO) signaling pathway, and (**d**) mitogen-activated protein kinases (MAPK) signal pathway. (**b**–**d**) show the z-scores based on the average of each expressed gene.

Analyses of the gene expression patterns of hatched larvae in the N-Ag-PVP-exposed group revealed an effect on genes related to tight junctions and the FOXO and MAPK signaling pathways (Figure 3a). Exposure to N-Ag-PVP increased the expression of major genes in the MAPK signaling pathway, thereby affecting cell proliferation and differentiation, and inducing inflammatory responses. The N-Ag-PVP exposure induced down-regulation of the cldna, cldnb, and oclua genes involved in intercellular signaling (Figure 3b). The HP-exposed group had similar regulation of the tight junction and MAPK signaling pathways to the control group. Moreover, Akt2l was down-regulated in all experimental groups compared with the control group, whereas foxo1a was up-regulated (Figure 3c). In addition, down-regulation of FOXO transcription factors, associated with homeostasis during environmental changes, stress, and inflammatory responses, is likely to impair blood glucose regulation by reducing glycolysis/gluconeogenesis, causing diabetes [55]. Furthermore, in the N-Ag-PVP-exposed group, up-regulation of mapk10 and mapk11 in the MAPK signaling pathway induced cell proliferation, cell differentiation, apoptosis, and inflammation, while down-regulation of tp53 prevented activation of the anticancer mechanism of p53, potentially leading to cancer (Figure 3d) [56].

N-Ag-PVP exposure up- or down-regulated genes involved in morphogenesis and development of important organs during embryonic development. N-Ag-PVP, N-Ag-PVP+HP, Hym176, and N-Ag-PVP+Hym176, all of which were absorbed into the cell, affected multiple signaling pathways within the cell. Tight junctions regulate the passage of ions and molecules in the epithelial and endothelial cell pathways [57]. Claudin, a cell membrane protein that constitutes a tight junction, determines intercellular ion selectivity [58]. However, N-Ag-PVP down-regulated claudin expression and decreased intercellular signal transduction. In addition, when the cell membrane proteins lose their fluidity, cellular structural integrity and signal transduction pathways may be impacted. In the absence of growth factor signaling or activation of Akt in the FOXO signaling pathway, FOXO up-regulates the target genes involved in stress resistance, metabolism, and apoptosis, which may impact lifespan and tumor growth [59]. The FOXO transcription factor remains inactive under normal conditions, but is activated to maintain homeostasis in response to environmental changes and stress; FOXO also inhibits insulin and growth factor signals [60]. In addition, genes in the MAPK signaling pathway regulate cell proliferation, differentiation, survival, inflammation, and apoptosis through stimulation of growth factors, cytokines, and stress and therefore play an important role in intracellular signal transduction [61].

**Figure 4:**
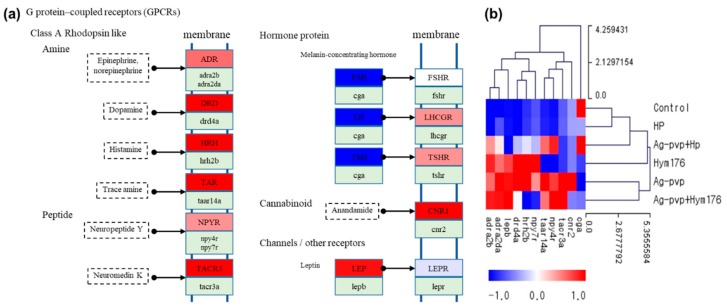
(**a**) Effects of N-Ag-PVP exposure on G-protein-coupled receptor (GPCR) gene expression in zebrafish larvae and (**b**) heatmap of GPCRs by z-score. N-Ag-PVP stimulated up-regulation of several GPCR genes. The z-score is based on the average of each expressed gene.

In the control and HP-exposed groups, similar expression patterns were observed for GPCRs, but up-regulation of amines and peptides of GPCRs and receptors for luteinizing hormone (LH) and thyroid stimulating hormone (TSH) were evident in the N-Ag-PVP-exposed group (Figure 4a). The control and HP-exposed groups had very similar expression patterns, except for the cga gene, where opposing results were obtained for the two groups (Figure 4b). GPCRs constitute a large proportion of the cell-surface receptors and regulate many cell functions, including cell proliferation, survival, and motility, and play a key role in tumor growth, angiogenesis, and metastasis [62]. The up- or down-regulation of GPCR-associated genes may inhibit receptor-mediated intercellular signaling [63,64].

**Figure 5:**
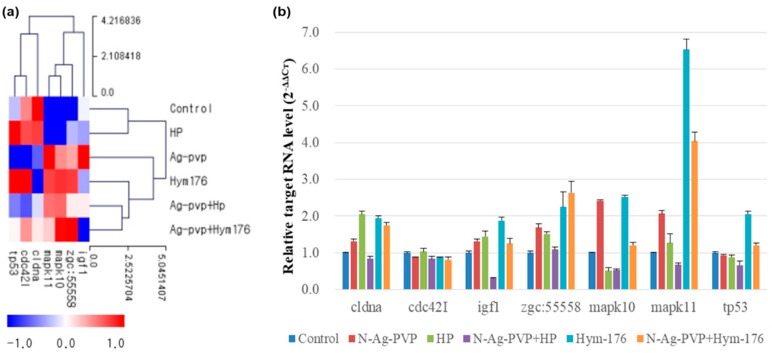
Comparison of expected and measured expression of key genes in zebrafish larvae exposed to N-Ag-PVP, HP, N-Ag-PVP+HP, Hym176, or N-Ag-PVP+Hym176. (**a**) Gene expression level by z-score and (**b**) relative target RNA level (2^−ΔΔCt^). The z-score is based on the average of each expressed gene (ΔCt = Target Ct − actb2 Ct, ΔΔCt = Target Sample ΔCt − control Sample ΔCt, 2^−ΔΔCt^ = Normalized gene amount of target group relative to target gene amount of control group).

The FC and qRT-PCR for the major genes in the intracellular signaling pathways were compared and shown in Figure 5. The Zgc:55558 was similarly expressed in all experimental groups, except for a 2.25- and 2.64-fold increase in the Hym176- and N-Ag-PVP+Hym176-exposed groups, respectively. In addition, the expression of Mapk10 increased more than two-fold in the N-Ag-PVP- and Hym176-exposed groups, while that of Mapk11 increased more than two-fold in the N-Ag-PVP-, Hym176-, and N-Ag-PVP+Hym176-exposed groups. The expression of Cdc42l was not significantly among the experimental groups.

Finally, N-Ag-PVP exposure caused an abnormal appearance in the zebrafish embryos, but co-incubation with HP or Hym176 tended to reduce the toxicity by forming aggregates with N-Ag-PVP. Therefore, additional studies investigating the formation of aggregates with nanomaterials are needed. In addition, further research addressing the genetic effects of nanomaterials is needed. It is expected that proteins and peptides derived from Hydra may be used as bio-derived materials for the capture and treatment of nanomaterials released into ecosystems.

## 4. Conclusions

We investigated and attempted to reduce the toxicity of N-Ag-PVP using the bioderived materials HP and Hym176. N-Ag-PVP and HP showed changes in surface charge and particle size after bonding, and N-Ag-PVP+HP formed agglomerates of 200 nm or more by electrostatic bonding. The N-Ag-PVP exposed group showed a low hatching rate (41.7 ± 2.9%) and high abnormality rate (27.8 ± 4.8%), including various forms such as inflammation, edema, and tail and kidney morphology in hatched fry. The group exposed to HP showed aggregated N-Ag-PVP to reduce toxicity. However, N-Ag-PVP+Hym176 exposure group showed a high rate of abnormality (39.4 ± 5.2%). In the results of the mRNA seq analysis, the gene expression pattern of the HP exposure group was different from those of the N-Ag-PVP, N-Ag-PVP+HP, Hym176, and N-Ag-PVP+Hym176 exposed groups. N-Ag-PVP has the potential to be directly responsible for abnormalities during the early developmental stage of the embryo, with variations in morphogenesis and development of key organs (heart, eyes, nerves, etc.). However, there was no genetic toxicity in the HP exposure group. HP is not toxic and has a tendency to reduce the ecotoxic and genotoxic effects of N-Ag-PVP. As a result, HP, a natural substance, shows potential as a candidate material for reducing the toxicity of nanomaterials.

## Figures and Tables

**Table 1:** Primer of key genes involved in the signaling pathway selected for qRT-PCR.

Gene Name	Gene Symbol	Function	Accession No.	Primer Sequences (5′ → 3′)
claudin a	cldna	structural molecule activity	NM _131762	(F) TGTGGCAAGTCACTGCTTTT (R) CACGCAACTCATCCAAATTC
cell division cycle 42, like	cdc42l	GTP binding, GTPase activity, nucleotide binding, protein kinase binding	NM _199865	(F) GGCAGGAAGACTACGACAGA (R) GGAGGAAGGAGAAACAACTGA
insulin-like growth factor 1	igf1	Growth factor activity, hormone activity, insulin-like growth factor receptor binding	NM _131825	(F) GTGGACGAATGCTGCTTTCA (R) CTGTCTTCACAGGCGCACAA
zgc:55558	zgc:55558	GTP binding, GTPase activity, nucleotide binding,	NM _200258	(F) TTTTTACACCCCCATCCTTT (R) GGTCTCGTGTGCACAGACAT
mitogen-activated protein kinase 10	mapk10	ATP binding, JUN kinase activity, MAP kinase activity, kinase activity, nucleotide binding, protein kinase activity, protein serine/threonine kinase activity, transferase activity	NM _001037701	(F) TCGAGGAGAGAACAAAGAATGG (R) AGGCTCTCGCTGCTGTTCAC
mitogen-activated protein kinase 11	mapk11	ATP binding, MAP kinase activity, kinase activity, nucleotide binding, protein kinase activity, protein serine/threonine kinase activity, transferase activity	NM _001002095	(F) CAGTACTGCCCTCTCCTTCTT (R) ATCGTCTCGTCTGGCTGAAC
tumor protein p53	tp53	DNA binding, DNA-binding transcription factor activity, metal ion binding, promoter-specific chromatin binding, protein binding, sequence-specific DNA binding, transcription regulatory region DNA binding,	NM _001271820	(F) TGCGATACATGTGATCCATT (R) CAGTGTCCAGCAACAAAGGT
actin, beta 2	actb2	ATP binding, nucleotide binding	NM _181601	(F) GACTCAAACTGCGCAGAGAA (R) AGTCAAGCGCCAAAAATAAC

**Table 2:** Analyses of zeta potential and DLS changes of N-Ag-PVP and N-Ag-PVP + HP.

	N-Ag-PVP	N-Ag-PVP+HP
Zeta potential (mV)	−22.84 ± 0.16	−5.54 ± 0.80
Diameter (nm)	285.50 ± 16.40	534.40 ± 122.05

**Table 3:** Comparison of the number of genes with differences in expression level.

		Total Genes	Up-Regulated	Down-Regulated
N-Ag-PVP	vs. Control	957 (6.61%)	377	580
HP	vs. Control	2686 (18.56%)	1066	1620
N-Ag-PVP+HP	vs. Control	928 (6.41%)	379	549
Hym176	vs. Control	1229 (8.49%)	545	684
N-Ag-PVP+Hym176	vs. Control	1284 (8.87%)	632	652
N-Ag-PVP+HP	vs. HP	2879 (19.89%)	1652	1227
N-Ag-PVP+Hym176	vs. Hym176	164 (1.13%)	112	52
N-Ag-PVP+Hym176	vs. N-Ag-PVP+HP	353 (2.44%)	202	151
N-Ag-PVP+HP	vs. N-Ag-PVP	110 (0.76%)	49	61
N-Ag-PVP+Hym176	vs. N-Ag-PVP	552 (3.81%)	332	220

**Table 4:** Genes showing toxicity reduction effects classified according to GO of morphogenesis.

Gene Description	Gene Symbol	Regulation Profile and Ratio(Fold change)				
		N-Ag-PVP /Control	HP /Control	N-Ag-PVP+HP /Control	Hym176 /Control	N-Ag-PVP +Hym176 /Control
**Heart morphogenesis (GO:0003007)**						
LIM domain 7b	lmo7b	2.708	0.743	1.415	2.264	1.425
fibronectin 1b	fn1b	2.179	1.643	1.723	3.874	5.859
myosin binding protein C, cardiac	mybpc3	0.357	1.706	0.569	0.493	0.959
**Fin morphogenesis (GO:0033334)**						
Sec23 homolog A, COPII coat complex component	sec23a	0.448	1.369	0.709	0.588	0.832
procollagen-lysine, 2-oxoglutarate 5-dioxygenase 1a	plod1a	0.441	0.869	0.611	0.462	0.665
**Embryonic organ morphogenesis (GO:0048562)**						
LIM domain 7b	lmo7b	2.708	0.743	1.415	2.264	1.425
lumican	lum	0.491	1.359	0.620	0.701	0.625
Sec23 homolog A, COPII coat complex component	sec23a	0.448	1.369	0.709	0.588	0.832
**Eye morphogenesis (GO:0048592)**						
intraflagellar transport 80 homolog (Chlamydomonas)	ift80	2.179	0.722	1.356	1.416	1.203
tubulin, gamma complex associated protein 4	tubgcp4	2.017	0.698	1.563	1.177	0.896
lumican	lum	0.491	1.359	0.620	0.701	0.625
adiponectin receptor 1b	adipor1b	0.444	0.924	0.620	0.271	0.371

## Supplementary Materials

The following are available online at https://www.mdpi.com/2079-4991/9/9/1210/s1, Figure S1: **A**, FE-SEM analysis of (**a**) N-Ag-PVP, (**b**) HP and (**c**) N-Ag-PVP+HP. * Red wedge is N-Ag-PVP; **B**, XRD patterns of (**a**) N-Ag-PVP and (**b**) N-Ag-PVP+HP; **C**, High resolution-raman analysis of N-Ag-PVP, N-Ag-PVP+HP and HP. Figure S2: Condition of embryos (1.5 hpf) and media immediately after exposure about (a) Control, (b) N-Ag-PVP, (c) HP, (d) N-Ag-PVP+HP, (e) Hym176 and (f) N-Ag-PVP+Hym176(c, chorion). Figure S3: The effects of the N-Ag-PVP with Hydra materials (HP, Hym 176) and pure N-Ag-PVP on the development of zebrafish. Embryos were exposed to (a) Control, (b) N-Ag-PVP(1 mg/L), (c) HP(4 mg/L), (d) N-Ag-PVP+HP(1 or 4 mg/L), (e) Hym176(4 mg/L) and (f) N-Ag-PVP+Hym176(1 or 4 mg/L). These images show exposed zebrafish at 72 hpf. Abbreviations: i, inflammation; t, tail; n, notochord; e, edema; k, kidney; y, yolk sac., Figure S4: Hierarchical clustering analyzed by z-score of differentially expressed genes in zebrafish larva exposed to each experimental group (Fold change: ≥2, ≤0.5; Normalized RC (log2): ≥5). The z-score is based on the mean of each expressed gene.
