# Supplementary material for: Reduction in Toxicity of Nano-Ag-Polyvinyl-pyrrolidone Using Hydra Proteins and Peptides during Zebrafish Embryogenesis
Source: Nanomaterials (Basel). 2019 Aug 27;9(9):1210. doi: 10.3390/nano9091210 (PMC6780337; doi:10.3390/nano9091210)
Supplement: Supplementary file 1 [file nanomaterials-09-01210-s001.pdf]

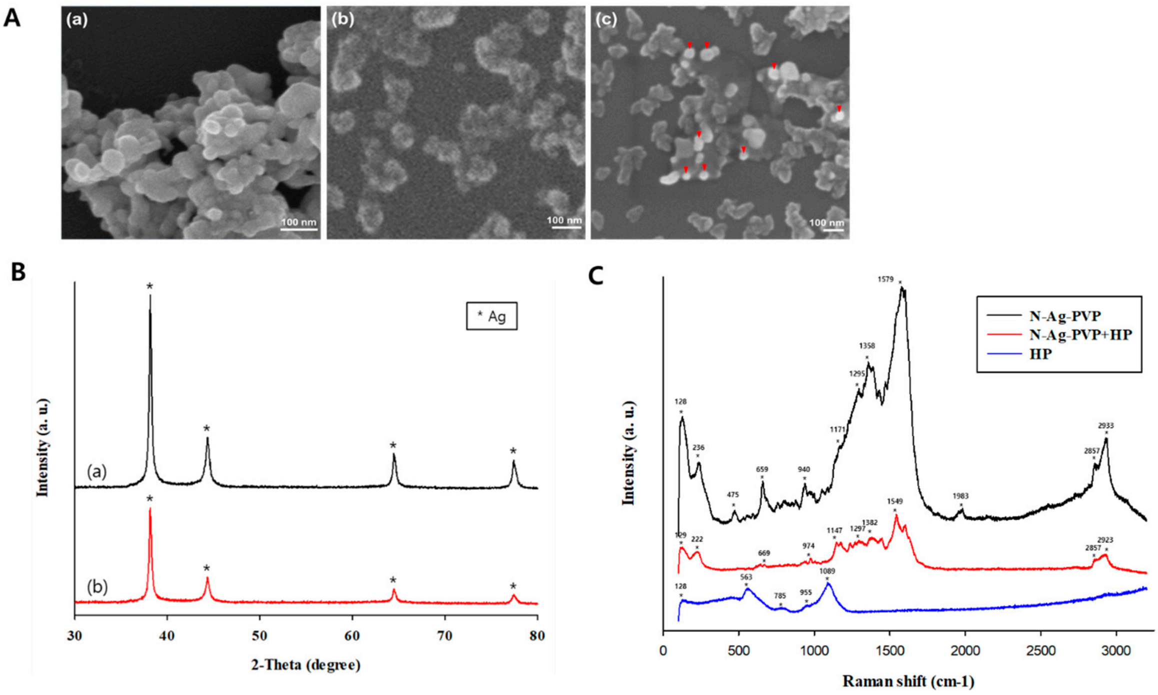

Figure S1. A, FE-SEM analysis of (**a**) N-Ag-PVP, (**b**) HP and (**c**) N-Ag-PVP+HP. * Red wedge is N-Ag-PVP; **B**, XRD patterns of (**a**) N-Ag-PVP and (**b**) N-Ag-PVP+HP; **C**, High resolution-raman analysis of N-Ag-PVP, N-Ag-PVP+HP and HP.

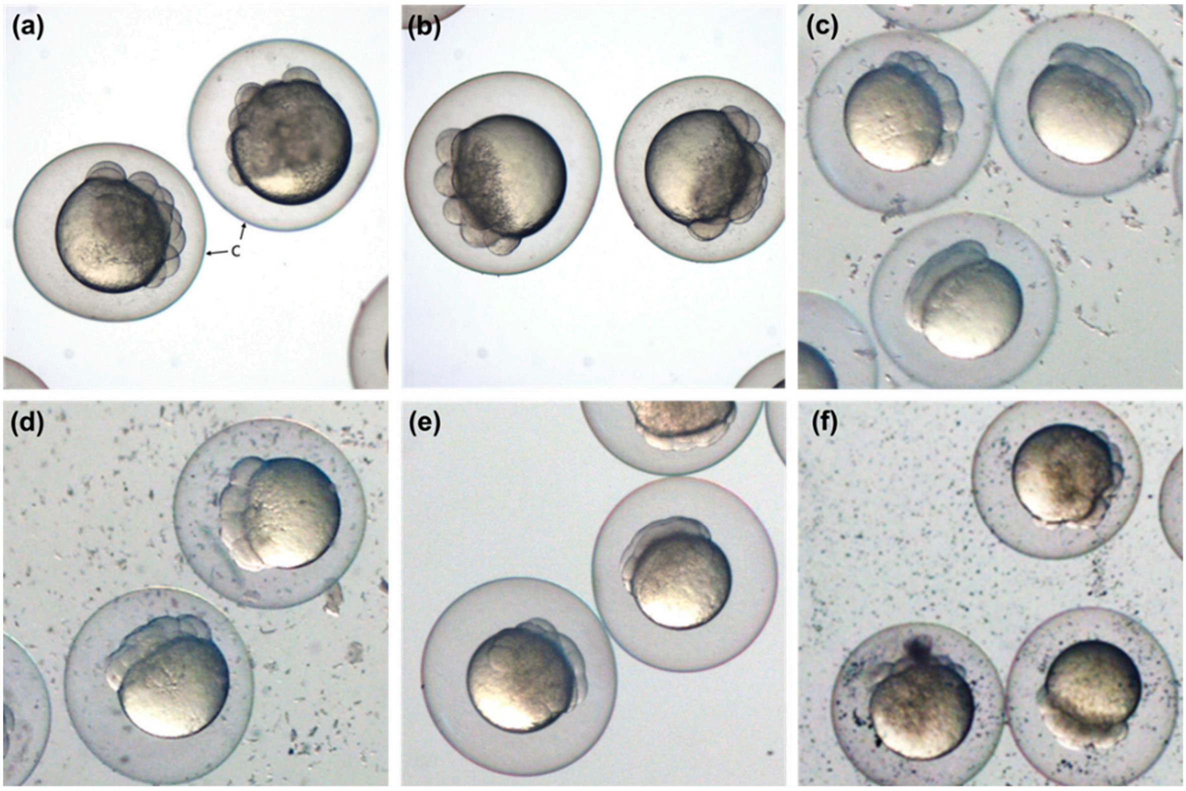

Figure S2. Condition of embryos (1.5 hpf) and media immediately after exposure about (**a**) Control, (**b**) N-Ag-PVP, (**c**) HP, (**d**) N-Ag-PVP+HP, (**e**) Hym176 and (**f**) N-Ag-PVP+Hym176 (c, chorion).

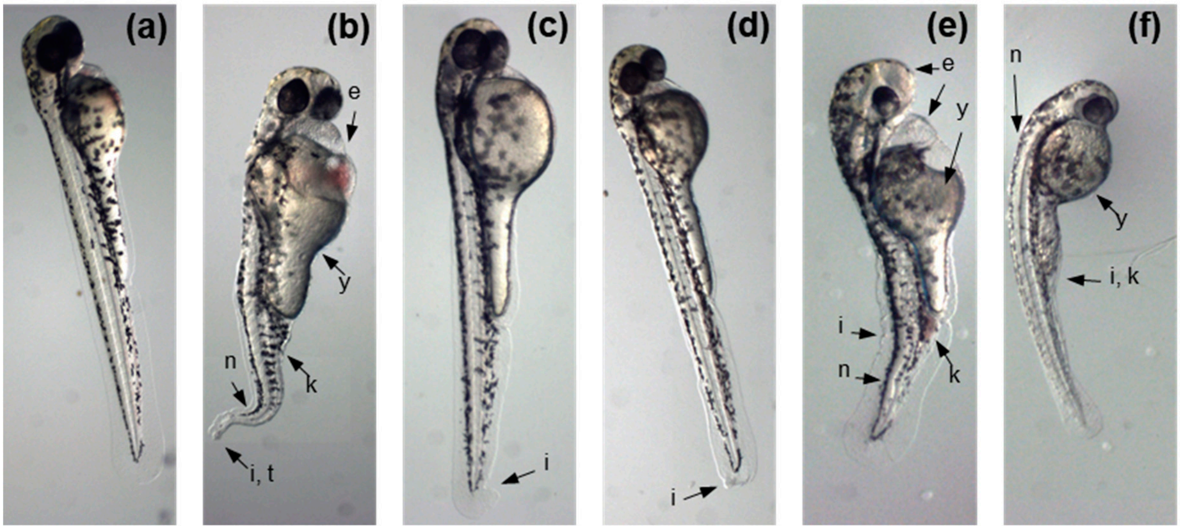

Figure S3. The effects of the N-Ag-PVP with Hydra materials (HP, Hym 176) and pure N-Ag-PVP on the development of zebrafish. Embryos were exposed to (a) Control, (b) N-Ag-PVP(1 mg/L), (c) HP(4 mg/L), (d) N-Ag-PVP+HP(1 or 4 mg/L), (e) Hym176(4 mg/L) and (f) N-Ag-PVP+Hym176(1 or 4 mg/L). These images show exposed zebrafish at 72 hpf. Abbreviations: i, inflammation; t, tail; n, notochord; e, edema; k, kidney; y, yolk sac.

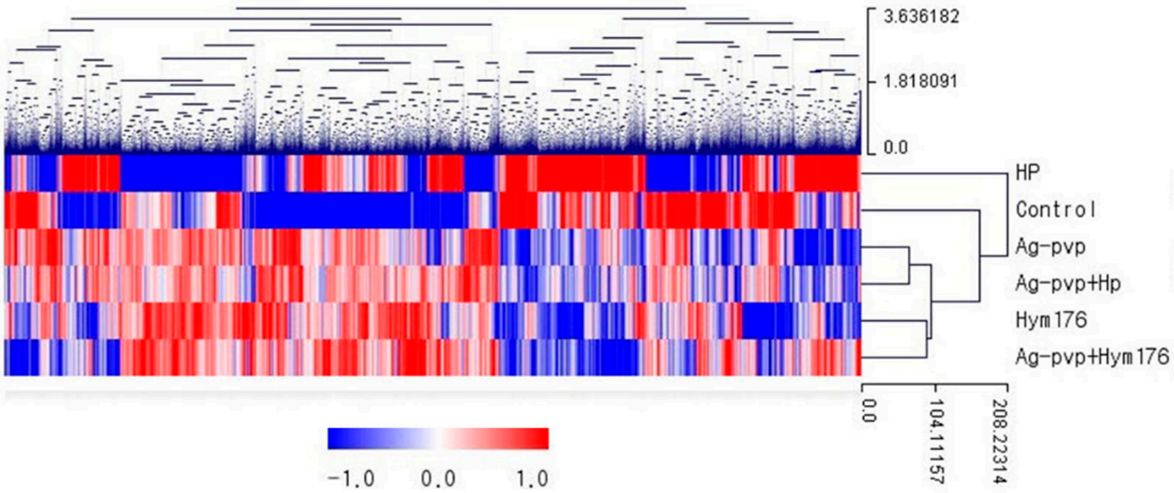

Figure S4. Hierarchical clustering analyzed by z-score of differentially expressed genes in zebrafish larva exposed to each experimental group (Fold change: ≥2, ≤0.5; Normalized RC (log2): ≥5). The z-score is based on the mean of each expressed gene.
